# Comparison of Acute Kidney Injury During Treatment with Vancomycin and either Piperacillin-Tazobactam or Meropenem

**DOI:** 10.51894/001c.6440

**Published:** 2017-12-19

**Authors:** John M. Cannon, Richard W. Douce, Erin R. Grubbs, Christopher B. Wills, Asam Khan, Elizabeth M. Schmidt, Michael S. Wang

**Affiliations:** 1 Lakeland Health Department of Pharmacy, Clinical Pharmacist, St. Joseph, MI; 2 Lakeland Health Department of Medicine, Core Faculty, St. Joseph, MI, Lakeland Health Internal Medicine Resident, PGY-3 Resident, St. Joseph, MI; 3 Lakeland Health Internal Medicine Resident, PGY-3 Resident, St. Joseph, MI; 4 Lakeland Health Internal Medicine, Program Director, St. Joseph, MI

**Keywords:** nephrotoxicity, meropenem, piperacillin-tazobactam, vancomycin

## Abstract

**CONTEXT:**

Empiric antibiotics are often required in hospitalized patients with serious infections who may be septic and at risk for drug resistant organisms. The purpose of this study was to evaluate the observed incidence of acute kidney injury (AKI) in a sample of adult patients receiving either piperacillin-tazobactam and vancomycin or meropenemvancomycin for at least 72 hours.

**METHODS:**

Single-center, retrospective matched cohort at a 200-bed Regional Community Medical Center. Adult patients were included in the sample if they were without preexisting renal dysfunction and admitted over an 18-month time period to receive either the combination of piperacillin-tazobactam and vancomycin or meropenem-vancomycin. Sample patients were evaluated for AKI. This condition was defined by the authors as an increase in serum creatinine of 0.5mg/ml or an increase of 50% above baseline during the duration of antibiotic treatment.

**RESULTS:**

A total of 266 patients receiving either combination of antibiotics were evaluated for AKI. The incidence of AKI was significantly higher in the piperacillin-tazobactam and vancomycin group (n = 74/292, 25%) compared with the meropenem-vancomycin group (n=8/74, 9.5%, p=0.008).

**CONCLUSIONS:**

The results of this study suggest that the combination of piperacillin-tazobactam and vancomycin is associated with an increased incidence of AKI. Higher vancomycin trough concentrations were associated with increased risk for development of AKI.

## INTRODUCTION

Empiric antibiotics are often required in hospitalized patients with serious infections who may be septic and at risk for drug resistant organisms.^1,2^ As sepsis-associated mortality is reduced with earlier antibiotics initiation, initial broad spectrum combination antibiotic therapy is often essential.^1,3^ These regimens typically include antibiotics that require coverage for both methicillin resistant *Staphylococcus aureus* (MRSA) and gram negative bacteria including *Pseudomonas aeruginosa*.^1,4^ Combination therapy in these instances may often include vancomycin, which covers MRSA, and an extended-spectrum beta lactam agent such as piperacillin-tazobactam (brand name Zosyn).

Both of these drugs have been in use for many years. It has been recognized that vancomycin, which covers MRSA, can cause nephrotoxicity.^2^ Piperacillin-tazobactam is a beta lactam antibiotic and member of the penicillin family of antibiotics. It is a broad-spectrum antibiotic with a scope of activity that covers gram-positive organisms, gram-negative organisms and anaerobes, but does not cover MRSA.^5^ Gram-positive organisms include *staphylococcus* and *streptococcus* species of bacteria, while *Escherichia coli*, Klebsiella species, and *Pseudomonas aeruginosa* predominate among gram-negative isolates.^6^

Both types of bacteria can also cause severe sepsis, although studies have demonstrated bacteremia (bacteria in the bloodstream) in gram negative patients at a higher frequency than gram positive patients­­­ among septic patients.^6^ In terms of kidney failure, piperacillin-tazobactam monotherapy has been shown to elevate creatinine, a marker of kidney function, in only 0.4% of patients.^5^ It has only recently been demonstrated that the combination of piperacillin-tazobactam with vancomycin can further increase the risk of nephrotoxicity.^7–18^

Renal function is often measured by serum creatinine, and an acute increase often represents kidney failure.^19^ It has been previously shown that an increase in serum creatinine (SCr) of >/= 0.5mg/dl is associated with increased odds of death by 6.5 times, an average increased length of stay (LOS) of 3-5 days, and increased hospital costs of $7500.^20^

Due to the reported nephrotoxicity associated with the vancomycin and piperacillin-tazobactam combination, alternative regimens may be necessary. A possible alternative initial therapy for sepsis that would cover MRSA and *pseudomonas aeruginosa* might include vancomycin and meropenem (brand name Merrem).^7^ Meropenem is a carbapenem and part of the beta lactam family of antibiotics. It has a spectrum of coverage similar to piperacillin-tazobactam and has a rate of creatinine increase of less than 1%.^21^ To our knowledge, only two other published studies have examined the rate of acute kidney injury (AKI) occurring when a carbapenem is combined with vancomycin across multiple disease states.^7,14^

The purpose of this retrospective study was to investigate whether the risk of AKI was greater in a convenience sample of hospitalized patients receiving the combination of piperacillin-tazobactam with vancomycin verses meropenem with vancomycin.

## METHODS

### Study Setting

This study was conducted at Lakeland Regional Medical Center, a 200-bed hospital with both teaching and non-teaching inpatient services in St. Joseph, Michigan. It is a Level 3 Trauma Center with an adult intensive care unit, a medical, oncological, post-surgical, orthopedic, neurological, and pediatric unit. Before data collection, the study was approved by the Lakeland Regional Medical Center Institutional Review Board.

### Study Design and Population

This was a single-center, retrospective, matched cohort study comprised of patients admitted to Lakeland Regional Medical Center between March 2012 and October 2014. The authors obtained pharmacy logs to review patients who had received Piperacillin/tazobactam-vancomycin (PT-vancomycin) or meropenem and vancomycin. The typical dose of piperacillin-tazobactam was either 3.375 grams, 4.5 grams, or 4.5 grams every six hours, depending on the patient’s treated condition, as higher doses are recommended for hospital-acquired pneumonia. The typical meropenem dose was one gram every eight hours.

The authors based any dosage adjustments on individual factors such as age and renal function. Vancomycin dosing was per discretion of the provider or prescribed as ‘pharmacy to dose’. When ordered as pharmacy to dose the clinical pharmacists monitored vancomycin levels, renal function, and adjusted the doses accordingly.

Patients were included in the analytic sample if they were over 18 years old, without pre-existing renal dysfunction, and received a minimum of 72 hours of a combination of either PT-vancomycin or meropenem with vancomycin for any indication. For both cohort groups, providers started concurrent therapy with required 48 hours of vancomycin initiation. At least one Vancomycin trough level and three serum creatinine levels were required during their hospital stay.

The authors excluded patients from the study if they possessed any signs of pre-existing renal dysfunction. This was defined as a baseline serum creatinine concentration of >1.5mg/dL (a marker of kidney function), a history of renal replacement therapy, or recent AKI within the past six months. Patients were excluded if they had not received at least 72 hours of concomitant antibiotics or if they received both piperacillin/tazobactam and meropenem at any point during their hospitalization. If a patient had multiple admissions during the study period, only the first hospitalization was used for the purposes of this study.

### Study Outcome

The primary result was the difference in the incidence of nephrotoxicity in patients either on PT-vancomycin or meropenem-vancomycin. This was defined as an increase in serum creatinine of 0.5mg/mL or an increase of 50% above the baseline. Other variables that were examined included: any concomitant use of nephrotoxic medications, advanced age, diabetes, and elevated Charlson Comorbidity Index (CCI) score. A CCI score was formulated to classify comorbid sample patients according to their risk of death from those diseases at the time of inclusion into the study.^22^

### Data Collection

The authors captured study data using the Lakeland Health electronic medical record system. The authors included patient data concerning:

- age
- gender
- height
- weight
- intensive care unit admission vs. all other units
- serum creatinine (on three separate dates if possible)
- number of days to increased serum creatinine if applicable
- antibiotic start and stop dates
- vancomycin serum concentration levels and dates drawn
- concomitant nephrotoxins (e.g., aminoglycosides, amphotericin, acyclovir, NSAIDS, loop diuretics)
- complete blood count on antibiotic initiation
- targeted medical condition diagnoses used to calculate CCI
- infectious disease diagnosis.

Statistical methods included the use of the chi-square test of association, Fisher’s Exact Probability Test, and ANOVA.^23^ Computations were performed via the VassarStats computational software (MW,RD).^24^

## RESULTS

A total of 454 patients who received piperacillin-tazobactam and vancomycin during the study period were first identified. Within this sample, 162 patients were excluded, either due to baseline renal insufficiency, prolonged use of multiple classes of antibiotics, and/or missing data concerning dates. Excluded patients were similar in age to the study population in the piperacillin-tazobactam and vancomycin group (65.6 vs. 65.3, p = 0.840), but slightly older in the meropenem and vancomycin group (67.5 vs. 60.3, p = 0.014). A total of 292 patients were treated with a combination of piperacillin-tazobactam and vancomycin and 74 patients were treated with meropenem-vancomycin.

Patients in the piperacillin-tazobactam and vancomycin group were slightly older (65.3 vs. 60.3 years, p = 0.013) on average than the meropenem-vancomycin group. (Table 1) Patients in the meropenem-vancomycin group were more likely to have been in the intensive care unit (31.1% vs. 15.8%, p = 0.003) and to have received contrast dye than the piperacillin-tazobactam and vancomycin group (50% vs. 31.2%, p = 0.003). There were no statistically significant differences found between sample subgroups in terms of gender, history of hypertension, elevated vancomycin trough, NSAID use, diuretic use, or ACE inhibitor use. The incidence of AKI was higher in the piperacillin-tazobactam group than the meropenem-vancomycin group (25% vs. 9.5%, p = 0.008).

**Table 1:** Comparison of Piperacillin/tazobactam with Vancomycin Versus Meropenem with Vancomycin

	**Piperacillin-tazobactam**	**Meropenem**	p-value *
	**(N=292)**	**(N=74)**	
**Age**	65.3+15.5	60.3+16.1	**0.013^**
**Gender**	168 (60.0%)	35 (47.3%)	0.07
**ICU during hospital stay**	46 (15.8%)	23 (31.1%)	**0.003**
**Baseline creatinine (mg/dL)**	0.92+0.28	0.88+0.32	0.34 ^
**Vancomycin trough > 15 mcg/mL**	130 (44.5%)	30 (40.5%)	0.60
**Vancomycin trough >20 mcg/mL**	64 (21.9%)	21 (28.4%)	0.47
**WBC (mm^3^)**	14.0+6.8	13.0+9.3	0.4 ^
**Diabetic**	123 (42.1%)	36 (48.6%)	0.24
**NSAID**	30 (10.3%)	5 (6.8%)	0.39
**ACE Inhibitor**	101 (34.6%)	26 (35.1%)	0.48
**Loop diuretic**	138 (47.3%)	40 (54.1%)	0.30
**IV contrast**	91 (31.2%)	37 (50%)	**0.003**
**Charlson score**	2.8+2.4	2.2+1.9	0.06 ^
**Creatinine > 1.5 x baseline or 0.5 increase**	74 (25%)	8 (9.5%)	**0.008**

*p-value calculated by two-tailed Fischer exact probability unless otherwise specified

^p-value calculated by Chi-square test

Table 2 compares patients with renal toxicity versus no renal toxicity in the two cohorts. In the piperacillin-tazobactam and vancomycin group, there were no differences in average age (65.7 vs. 64.6 years, p = 0.62), gender (60.8% male vs. 55.6% female, p = 0.27), or ICU stay (20.3% vs. 14.2%, p = 0.27). Patients whose vancomycin troughs, which are steady state concentration levels, were >20 mcg/mL were more likely to have renal insufficiency (41.9% vs. 15.6%, p = 0.01). Patients whose vancomycin troughs were > 15 mcg/mL were also more likely to have renal insufficiency (62.2% versus 38.1%, p = 0.001).

**Table 2:** Patients with Renal Insufficiency Versus No Renal Insufficiency

	**Renal insufficiency**	**No renal-insufficiency**	p-value *
***Piperacillin-tazobactam***			
**Age**	65.7	64.6	0.62 ^
**Gender**	45/74 (60.8% male)	122/218 (55.6%)	0.50
**ICU**	15/74 (20.3%)	31/218 (14.2%)	0.27
**Baseline creatinine (mg/dL)**	0.92	0.90	0.71 ^
**Vancomycin trough > 15 mcg/mL**	46/74 (62.2%)	83/218 (38.1%)	**0.01**
**Vancomycin trough >20 mcg/mL**	31/74 (41.9%)	34/218 (15.6%)	**<0.001**
			
***Meropenem***			
**Age**	60.8	58.3	0.73 ^
**Gender**	5/8 (62.5%)	30/66 male (45.5%)	0.46
**ICU**	4/8 (50%)	19/66 (28.8%)	0.23
**Baseline creatinine (mg/dL)**	1.16	0.82	**0.009 ^**
**Vancomycin trough > 15**	6/8 (75%)	24/66 (36.4%)	0.055
**Vancomycin trough >20**	4/8 (50%)	17/66 (25.8%)	0.21

*p-value calculated by two-tailed Fischer exact probability unless otherwise specified

^p-value calculated by Chi-square test

Comparing patients with renal toxicity versus no renal toxicity in the meropenem-vancomycin group, there were no differences in average age (60.8 vs. 58.3, p = 0.73), gender (62.5% male vs. 45.5%, p = 0.46), or ICU stay (50% vs. 28.8%, p = 0.23). The average baseline creatinine was higher in the renal toxicity group (1.16 vs. 0.82, p = 0.009). There was a trend toward elevated vancomycin trough, trough > 15 mcg/mL in the renal toxicity group (75% vs. 36.4%, p = 0.055), although this was not statistically significant. Overall, the risk of renal failure with vancomycin in combination with meropenem vs. piperacillin-tazobactam was 0.3571 (95% CI 0.1637 to 0.7787, p=0.008), as indicated in Table 3.

**Table 3:** Risk of Renal Failure with Vancomycin in Combination with Meropenem Versus Piperacillin-Tacobactam

**Odds ratio**	0.3571
**95% CI**	0.1637 to 0.7787
**z statistic**	2.589
**Significance level**	**P=0.008 ^**

^ p-value calculated by two-tailed Fischer exact probability

## DISCUSSION

Our study compared the incidence of AKI between an extended beta lactam, piperacillin-tazobactam plus vancomycin with a carbapenem (meropenem) plus vancomycin. Our analysis found that the use of the piperacillin-tazobactam and vancomycin combination was associated with a statistically significant increased incidence of AKI compared to the meropenem-vancomycin combination. This finding occurred despite the observation that the patients in the meropenem and vancomycin group were significantly more likely to have been in the intensive care unit, had a higher average creatinine and had received contrast dye.

Among patients who developed renal insufficiency on piperacillin-tazobactam and vancomycin, the biggest predictor of renal insufficiency was an elevated vancomycin trough. More specifically, patients with troughs over 15 and 20 mcg/mL were more likely to have developed renal insufficiency. However, the authors did not find an association with other nephrotoxic agents and renal insufficiency. Among those patients who had developed nephrotoxicity in the meropenem-vancomycin group, there was a trend toward elevated trough levels, although our sample size was too small to test this pattern for possible statistical significance.

Since vancomycin is a common component of empiric dual antibiotic regimens where MRSA, gram negative and anaerobic coverage is sought, examining incidence rates of AKI with various vancomycin combination therapies is important. In this study, AKI incidence was significantly greater in the vancomycin and piperacillin-tazobactam group compared to the vancomycin-meropenem group (table 1).

To our knowledge, this is the third published study examining the differential rates of AKI with these two commonly used antibiotic combinations across multiple disease states. A single center retrospective study comparing the nephrotoxicity of vancomycin plus meropenem in 75 patients with vancomycin plus piperacillin-tazobactam in 108 patients was unable to demonstrate statistical significance.^7^ However, the results of another prospective study demonstrated statistical significance in comparing AKI in a group that had received vancomycin plus either meropenem or cefepime with a group that received PT-vancomycin.^14^

The specific mechanism of action linking increased incidence of AKI with the combination of PT-vancomycin remains unknown. The two identified mechanisms of AKI, namely acute interstitial nephritis with piperacillin-tazobactam and direct cellular necrosis with vancomycin may interact either additively or synergistically. Additional studies are needed to further elucidate and validate this hypothesis.

Despite the risk of nephrotoxicity associated with piperacillin-tazobactam and vancomycin, switching to alternative regimens that cover MRSA and *Pseudomonas* carries significant AKI risks as well. Previous studies have demonstrated cefepime and vancomycin as carrying less of a risk for nephrotoxicity.^12,14^ However, cephalosporins have been demonstrated to carry a higher risk of contributing to Clostridium difficile infections.^24^ Although our study results demonstrated a decreased risk of renal insufficiency in the meropenem-vancomycin group, meropenem should still be used with caution due to a potential rise in organisms carrying *Klebsiella* producing carbapenemases (KPC).^25^ The rationale for this being that KPC organisms cause resistance in almost all beta-lactam antibiotics.^25^ A future study examining piperacillin-tazobactam and other anti-MRSA antibiotics (such as linezolid or daptomycin) should be considered. Finally, an additional consideration would be to encourage discontinuation of vancomycin when possible, as IDSA antimicrobial stewardship guidelines encourage narrowing of therapy, if clinically appropriate.^26^

This study illustrates the challenges associated with antimicrobial usage in complex clinical scenarios. Antibiotic stewardship has become increasingly important in the age of multi-drug resistant infections. Strategies such as de-escalation of antibiotic therapy and reducing unnecessary antibiotic usage are also important in reducing unintended side effects.^26^ Alternative strategies of piperacillin-tazobactam have also been discussed and could potentially have an effect on patients’ renal function.^25^

This study has several limitations that are worth noting. Data were collected retrospectively from a convenience sample without the benefit of blinding from the electronic health record. Our retrospective design also made it difficult for us to ensure the comparability of the two study groups. In fact, we realized that there were likely confounding differences in disease severity and complexity between patients who received either the vancomycin-PT combination or the vancomycin-meropenem combination that also could have influenced renal function. Still, we attempted to control for potential confounders by including concomitant use of nephrotoxic drugs and illness severity (as reflected by the Charlson Comorbidity Index).

We believe that our findings, combined with those of previous studies,^10^ should prompt further investigation in this area primarily since the antibiotic combinations investigated here are so very commonly used in numerous hospitalized patients across the United States. Although the incidence of AKI was relatively low when vancomycin was solely used, patient receiving the combination of vancomycin and piperacillin-tazobactam had a significantly increased risk of developing AKI.

## CONCLUSIONS

In conclusion, this study demonstrated an increased risk of developing AKI with the combination of vancomycin and piperacillin-tazobactam compared to the combination of vancomycin and meropenem. Research groups have not yet definitively identified the specific mechanism underlying the increased incidence of AKI with the piperacillin-tazobactam and vancomycin combination regimen. Larger robust studies are required before national groups can formulate conclusive clinical guidelines with respect to use of vancomycin in combination with piperacillin-tazobactam.

### Conflict of Interest

The authors declare no conflict of interest.
